# Renin-a in the Subfornical Organ Plays a Critical Role in the Maintenance of Salt-Sensitive Hypertension

**DOI:** 10.3390/biom12091169

**Published:** 2022-08-24

**Authors:** Silvana G. Cooper, Lucas A. C. Souza, Caleb J. Worker, Ariana Julia B. Gayban, Sophie Buller, Ryosuke Satou, Yumei Feng Earley

**Affiliations:** 1 Departments of Pharmacology, and Physiology & Cell Biology, School of Medicine, University of Nevada, Reno, NV 89557, USA; silvanac@med.unr.edu (S.G.C.); lsouza@med.unr.edu (L.A.C.S.); c_worker@outlook.com (C.J.W.); arianaj@nevada.unr.edu (A.J.B.G.); 2 Center for Molecular and Cellular Signaling in the Cardiovascular System, University of Nevada, Reno, NV 89557, USA; 3 MRC Institute of Metabolic Science, University of Cambridge, Cambridge CB2 1TN, UK; sab236@medschl.cam.ac.uk; 4 Department of Physiology, Tulane University School of Medicine, New Orleans, LA 70112, USA; rsato@tulane.edu

**Keywords:** renin-angiotensin system, salt-sensitive hypertension, NAD(P)H oxidase, angiotensin receptor, autonomic control

## Abstract

The brain renin-angiotensin system plays important roles in blood pressure and cardiovascular regulation. There are two isoforms of prorenin in the brain: the classic secreted form (prorenin/sREN) encoded by renin-a, and an intracellular form (icREN) encoded by renin-b. Emerging evidence indicates the importance of renin-b in cardiovascular and metabolic regulation. However, the role of endogenous brain prorenin in the development of salt-sensitive hypertension remains undefined. In this study, we test the hypothesis that renin-a produced locally in the brain contributes to the pathogenesis of hypertension. Using RNAscope, we report for the first time that renin mRNA is expressed in the subfornical organ (SFO), where it is found in glutamatergic and GABAergic neurons. Notably, we found that renin mRNA was significantly elevated in the SFO and PVN in a mouse model of DOCA-salt–induced hypertension. To examine the functional importance of renin-a in the SFO, we selectively ablated renin-a in the SFO in renin-a–floxed mice using a Cre-lox strategy. Importantly, renin-a ablation in the SFO attenuated the maintenance of DOCA-salt–induced hypertension and improved autonomic function without affecting fluid or sodium intake. Molecularly, ablation of renin-a prevented the DOCA-salt–induced elevation in NADPH oxidase 2 (NOX2) in the SFO without affecting NOX4 or angiotensin II type 1 and 2 receptors. Collectively, our findings demonstrate that endogenous renin-a within the SFO is important for the pathogenesis of salt-sensitive hypertension.

## 1. Introduction

The renin-angiotensin system (RAS) plays an important role in the regulation of blood pressure (BP) as well as cardiovascular, metabolic, and body fluid homeostasis [1,2,3]. In addition to the classical circulating/endocrine RAS, the local/tissue RAS is also critical for hypertension and cardiovascular regulation [3,4,5,6,7,8]. It is now well established that most RAS components are expressed in the central nervous system (CNS) [3,6,9,10,11,12]. Moreover, accumulating evidence supports a vital role for the local brain RAS in BP regulation and the pathogenesis of hypertension. Neither renin, the rate-limiting enzyme of the classical RAS, nor angiotensin II (Ang II), the main bioactive peptide of the RAS, can penetrate the blood–brain barrier (BBB) under physiological conditions [13,14]. However, they can reach the brain by accessing the circumventricular organs. We recently reported that the (pro)renin receptor (PRR)—the receptor for renin and prorenin—plays a critical role in the neural regulation of BP, possibility through activation of endogenous brain prorenin [10,15]. However, direct evidence of a role for endogenous brain prorenin in BP regulation and hypertensin is lacking.

Two different forms of prorenin are encoded by alternate transcripts in the brain: the classical secreted form of prorenin (also called sREN), encoded by renin-a, and an intracellular form of prorenin (icREN), encoded by an alternative transcript starting at exon 1b, named renin-b [16,17]. Elegant studies performed over the last decade have established the importance of icREN in the brain [18,19,20,21,22,23,24]. Interestingly, deletion of icREN activates the brain RAS, increasing BP as well as inducing dipsogenic and metabolic effects, possibly by increasing renin-a/sREN expression or activity [18,19,20,21,22,23,24]. While these findings reinforce the potential functional importance of endogenous prorenin in the CNS, there is no direct evidence for the function of brain renin-a/sREN in hypertension. In the present study, we test the hypothesis that endogenous renin-a plays a regulatory role in DOCA-salt hypertension. To this end, we used sophisticated techniques, including RNAscope and droplet digital PCR, to characterize the expression of endogenous renin in the brain. We further used a novel renin-a–floxed mouse model in combination with in vivo telemetry recordings to determine the functional importance of renin-a in BP regulation and hypertension. 

## 2. Materials and Methods 

### 2.1. Animals

Renin-a–floxed mice (in a C57Bl/6J background) used in these studies were generated in Curt Sigmund’s laboratory [23]. A colony of these mice has been established and maintained in our laboratory at the University of Nevada, Reno. Mice were provided ad libitum access to tap water and regular chow before experiments and were maintained on a 12-h light-dark cycle. Both male and female mice, age 8–12 weeks, were used in experiments. All procedures were conducted in accordance with the National Institutes of Health Guide for the Care and Use of Laboratory Animals and were approved by the Institutional Animal Care and Use Committee and the Institutional Biosafety Committee at the University of Nevada, Reno. The use of adeno-associated virus (AAV) was approved by the Institutional Biosafety Committee at the University of Nevada, Reno.

### 2.2. DOCA-Salt Hypertension Model 

The DOCA-salt hypertension model was prepared as described previously [10,15,25]. In brief, mice were anesthetized with isoflurane and implanted subcutaneously with either a sham pellet or a deoxycorticosterone-acetate (DOCA) pellet (2.5 mg/g body weight) without nephrectomy. Mice were provided free access to regular tap water (Sham group) or 0.9% saline (DOCA-Salt treatment group). Both Sham and DOCA-Salt mice were fed regular chow ad libitum for up to 3 weeks. 

### 2.3. Droplet Digital PCR 

Mice were treated with either DOCA-salt or were sham-treated as described above for 21 days, and their brains were rapidly removed and flash frozen. Tissue micro-punches of the subfornical organ (SFO), paraventricular nucleus (PVN), arcuate nucleus (ARC), rostral ventral lateral medulla (RVLM), nucleus tractus solitarius (NTS), and area postrema (AP) were isolated under a cryostat (CM 1950; Leica, Buffalo Grove, IL, USA) according to The Mouse Brain Atlas (2nd Edition, Paxinos & Franklin). Copy numbers of total renin (renin-a + renin-b) mRNA in distinct cerebral areas were determined using Droplet Digital PCR (ddPCR) as previously described [26,27]. Total RNA was isolated using a commercially available RNA isolation kit (Qiagen, Valencia, CA, USA), and RNA concentration was quantified using a Nanodrop 2000 (ThermoFisher Scientific, Waltham, MA, USA). Performance of ddPCR was done on a Bio-Rad ddPCR system using the renin primers 5′-TAT CCC AAC AGG AGA GAC AA-3′ (forward) and 5′-GAC AAA GCC AGA CAA AAT GG-3′ (reverse) and the Taqman probe, 5′-FAM-AGT AAC CCT AGG CCA AGC CA-BHQ1-3′. Other reagents used to generate cDNA and quantify gene expression, including One-Step RT-ddPCR master mix, were purchased from Bio-Rad (Los Angeles, CA, USA). After droplet generation and PCR amplification, droplets were analyzed on a QX200 droplet reader, and gene cDNA copy numbers were determined using QuantaSoft analysis software (Bio-Rad). β-actin mRNA levels (primer/probe: Bio-Rad #10031252) were detected by ddPCR prior to analysis of other target genes to adjust for minor differences in RNA concentrations among samples. For each renin gene, the amount of total RNA in a PCR reaction was determined by a pilot ddPCR using serially diluted total RNA in which the determined amount was in the linear range. In this ddPCR analysis, normalizing renin mRNA levels to β-actin mRNA levels is not necessary because the evaluation is based on the absolute copy number. Therefore, data are expressed as copy numbers of target gene per nanogram total RNA, assessed using at least three biological replicates.

### 2.4. RNAscope In Situ Hybridization of Renin mRNA 

In situ hybridization of renin mRNA was performed using an RNAscope Multiplex Fluorescent Assay kit (ACD Inc., Newark, CA, USA), together with a specific probe complementary to a section of the mouse renin 1 (*Ren1*) sequence (NCBI Gene ID: 19701), designed by ACD (Cat No. 433461). A scrambled probe (Cat No. 563411), designed and synthesized by ACD, was generated from the *Ren1* probe sequence for use as a control. In addition, negative control hybridizations were performed using a probe targeting the bacterial gene, DapB (4-hydroxy-tetrahydrodipicolinate reductase), also from ACD (Cat No. 310043) (data not shown). To identify types of neurons, we used RNAScope probes (purchased from ACD) targeting genes encoding vesicular glutamate transporter 2 (vGlut2; NCBI Gene ID: 140919, Cat No. 319171) and vesicular inhibitory amino acid transporter (VIAAT/VGAT; NCBI Gene ID: 22348, Cat No. 319191). Astrocytes and microglia were identified by performing immunolabeling at the end of RNAScope using antibodies against the astrocyte marker, glial fibrillary acidic protein (GFAP) (1:500 GFAP, cat. no. ab-4674; Abcam, Cambridge, MA, USA), and the microglia marker, ionized calcium-binding adapter molecule 1 (Iba-1) (1:500, cat. no. NB100-1028; Novus, Littleton, CO, USA). 

For RNAscope analyses, wild-type C57Bl/6J mice (n = 4) were transcardially perfused first with 0.9% saline and then with 4% paraformaldehyde (PFA; Sigma-Aldrich, St. Louis, MO, USA). Thereafter, brains were immediately extracted and kept in 4% PFA for 24 h at 4 °C and then transferred to 30% sucrose and stored for an additional 24 h at 4 °C. Brains were then frozen at −20 °C in Tissue-Plus Optimal Cutting Temperature Compound (ThermoFisher Scientific) for 12 h before sectioning. Coronal sections (15-μm thick) were cut using a cryotome (Leica CM1950; Leica) at −20 °C and mounted onto Colorfrost Plus slides (Thermo Fisher Scientific). Sections were dried and stored at −80 °C until use. The standard RNAscope protocol for fixed frozen tissue (ACD User Manual 323100-USM) was followed, with minor modifications. A HybEZ II Hybridization System (ACD Inc.) was used to control incubation temperature. 

Slides were baked at 60 °C for 20 min, then fixed in PFA for 15 min, followed by dehydration with a graded series of ethanol (50%, 70%, and 100%) for 5 minutes each. Slides were then immersed in 3% hydrogen peroxide for 10 min and rinsed in distilled H_2_O. Target retrieval was performed by steaming slides (Hamilton Beach 37530A steamer; Hamilton Beach, Glen Allen, VA, USA) for 6 min, and then re-immersing slides in 100% ethanol for an additional 2 min. Slides were air dried at room temperature, and a hydrophobic barrier was drawn surrounding the mounted tissue, after which Protease III was applied, and slides were incubated for 30 min at 40 °C. After washing slides in wash buffer, the renin probe, scrambled probe, or negative control probe (DapB) was applied, and slides were incubated at 40 °C for 2 h. For amplification, AMP1 was added, and slides were incubated for 30 min, followed by a 30 min incubation with AMP2 and a 15 min incubation with AMP3 (catalog number 320851) at 40 °C, with thorough washes in 1X wash buffer between steps. After amplification, a horseradish peroxidase (HRP)-conjugated probe of interest was applied and incubated for 30 min at 40 °C. This was followed by a 15 min incubation at 40 °C with the fluorophore, fluorescein (1:800 dilution), and a subsequent 15 min incubation with an HRP blocker at 40 °C. Nuclei were counterstained with 4′,6-diamidino-2-phenylindole (DAPI), after which sections were coverslip mounted using Prolong Gold Antifade Mountant (ThermoFisher Scientific) and imaged using a confocal microscope (Fluoview FV3000; Olympus Life Sciences, Center Valley, PA, USA). 

For immunolabeling of GFAP and Iba-1, after RNAscope labeling was completed and prior to DAPI staining, sections were washed twice in PBS and blocked by incubating in PBS containing 0.3% Triton X-100 and 10% normal house serum (NHS) for Iba1 sections and 0.3% Triton X-100 and 10% normal goat serum (NGS) for GFAP sections for 1 h at room temperature. Sections in PBS containing 0.3% Triton X-100 and 2% NHS or 2% NGS were incubated overnight at 4 °C with goat anti-IBA-1 (1:500, cat. no. NB100-1028; Novus) or chicken anti-glial fibrillary acidic protein (GFAP) (1:500, cat. no. ab-4674; Abcam, Cambridge, MA, USA) primary antibodies. Sections were washed four times in PBS and incubated for 2 h at room temperature with Alexa 488-conjugated donkey anti-goat (1:1000; cat. no. A-11055; Thermo-Scientific, Waltham, MA, USA) or Alexa 488-conjugated goat anti-chicken (1:1000; cat. no. A-11039; Thermo-Scientific, Waltham, MA, USA) secondary antibodies, diluted in PBS containing 0.3% Triton X-100 and 2% NGS or diluted in PBS containing 0.3% Triton X-100 and 2% NHS. Nuclei were counterstained with DAPI (4′,6-diamidino-2-phenylindole), and sections were coverslip mounted using Prolong Gold Antifade Mountant (Thermo Fisher Scientific) and imaged using a confocal microscope (Fluoview FV3000; Olympus Life Sciences).

All images were acquired at a resolution of 1024 × 1024 dpi and taken at 20.0 μs/pixel. Laser power, voltage, gain, and offset were optimized and kept consistent for all slides, including negative control slides. For cell counting analysis, at least 4 sections from each mouse (from four C57/BL6 mice) covering anterior to posterior of the SFO were used for RNAscope as described above. Cells were considered to express the mRNA of interest if at least three visible transcripts, defined as an individual punctate dot, were observed surrounding a nucleus [28]. Individual cells were identified using DAPI. Each nucleus (stained with DAPI) that was surrounded by magenta puncta was considered a renin-positive cell. Nuclei surrounded by magenta (renin) and yellow (vGlut2) were considered renin-glutamatergic cells. Nuclei with surrounding magenta (renin) and cyan (VIAAT) puncta were considered Renin-GABAergic cells. Nuclei with magenta (renin), yellow (vGlut2), and cyan (VIAAT) puncta were considered renin-glutamatergic + renin-GABAergic cells. 

### 2.5. Nano-Injection of AAVs into the SFO

Mice were anesthetized using 4–5% isoflurane in 100% O_2_, flushed at 1 L/min for 2 min, and anesthesia was subsequently maintained using 0.75–1.5% isoflurane. The top of each mouse’s head was shaved and sterilized with alcohol wipes, after which the mouse was placed in a digital stereotaxic apparatus (Stoelting, Wood Dale, IL, USA) and held in place by ear bars secured just above the ear canal. An incision (~1 cm) in the skin along the top of the head was made to expose the skull. The skull was cleaned with 3% hydrogen peroxide using cotton swabs, after which holes were drilled into the skull at the stereotaxic coordinates, 0.2 mm posterior to Bregma at the midline and 3.0 mm dorsal. A 1 µL Neuros Hamilton syringe (Hamilton, Reno, NV, USA) attached to a UMP3 syringe pump (WPI, Sarasota, FL, USA) was fixed to the stereotaxic frame and used for AAV nano-injections. The syringe needle (32 gauge) was lowered to 3.3 mm from the surface of the skull, and the AAV solution was injected into the SFO in a total volume of 100 nL at a rate of 1 nL/s. Renin-a–floxed mice received injections of either AAV2-Cre-eGFP (to knock down prorenin) or control AAV2-eGFP virus (1.1 × 10^9^ viral genomes). The syringe needle was left in place for an additional 5 min before removing to prevent backflow of the virus through the needle track. The wound was sutured, and the mice were allowed to recover on a heating pad. AAVs (AAV2/1CMV-eGFP and AAV2/1CMV-Cre-eGFP) were purchased from the Viral Vector Core Facility at the University of Iowa. These AAVs use the human cytomegalovirus promoter, allowing for efficient recombinant expression of Cre recombinase and eGFP reporter. We previously characterized these AAV2s, reporting that they were expressed exclusively in neurons and not astrocytes or microglia [25]. 

### 2.6. Telemetric Measurement of BP, Heart Rate, and Autonomic Function in Conscious, Freely Moving Mice

Mice were anesthetized with isoflurane as described above and the oblique and tracheal muscles were separated to expose the left carotid artery [10,15,25,29]. The catheter of a radio telemetry transmitter (PA C-10; DSI, Harvard Bioscience Inc, St. Paul, MN, USA) was implanted into the left carotid artery and secured with a suture. The body of the radio transmitter was subcutaneously implanted in the right flank under the arm. Mice were allowed to recover from surgery for 10–14 days, and BP and heart rate (HR) were monitored throughout the protocol in conscious, freely moving mice using telemetry. Autonomic function was evaluated by measuring HR and BP following intraperitoneal (ip) injection of a β-blocker (propranolol; 5 mg/kg), a muscarinic receptor blocker (methylatropine; 1 mg/kg), and a ganglionic blocker (chlorisondamine; 6 mg/kg). Changes in HR in response to propranolol and methylatropine represent cardiac sympathetic and parasympathetic tone, respectively, and the reduction in BP in response to chlorisondamine reflects the sympathetic contribution to BP. Telemetry parameters were recorded 1 h prior to ip injections (baseline) and continuously for 2–3 h after injections. Peak changes in HR and BP in response to each pharmacological antagonist occurred within 30 min of administration, and were calculated and presented as ΔHR and ΔBP. 

### 2.7. Real-Time PCR

Renin-a–floxed mice were bilaterally injected with either AAV2-Cre-eGFP or AAV2-eGFP, as described above, and allowed to recover for 7 days. Mice were then treated with DOCA-salt (or sham treated) as described above for 7 or 14 days. Mice were sacrificed by cervical dislocation, and their brains were rapidly removed and flash frozen. Total RNA from tissue micropunches of the SFO was isolated using TRIzol reagent (Thermo Fisher) following the manufacturer’s protocols. Briefly, tissue was homogenized in 500 µL of TRIzol using a TissueMiser (Tekmar, Vernon, Canada) and incubated for 5 min at room temperature. Samples were centrifuged at 10,000× *g* for 5 min, and the supernatant was transferred to a fresh tube. Chloroform (100 µL) was added to each sample, after which samples were vortexed for 15 s and incubated at room temperature for 5 min. Samples were centrifuged at 12,000× *g* for 15 min, and the clear aqueous phase was transferred to a fresh tube. RNA was precipitated by adding 250 µL of isopropyl alcohol, and the samples were incubated for 10 min at room temperature. Samples were centrifuged at 12,000× *g* for 10 min and the supernatant was discarded. The RNA pellet was washed twice by briefly vortexing in 500 µL of 75% ethanol and centrifuging at 7500× *g* for 5 min. Ethanol was removed and the pellet was allowed to air dry for 10 min at room temperature. RNA was dissolved in 30 µL of DNAse/RNAse-free H_2_O (Invitrogen), and its concentration and purity were determined using a NanoDrop spectrophotometer (Thermo Fisher). Samples were stored at −80 °C. cDNA was reverse transcribed (RT) using an Applied Biosystems high-capacity cDNA reverse transcription kit (Thermo Fisher), and quantitative polymerase chain reaction (qPCR) was performed using Fast SYBR Green master mix (Thermo Fisher). mRNA levels of angiotensin receptors (AT_1a_R and AT_2_R) and NAD(P)H oxidases (NOX2 and NOX4) were determined using the ∆∆CT relative quantification method and normalized to β-actin, expressed as fold change. The primer pairs used are listed in Table 1. 

### 2.8. Statistical Analysis

Data are expressed as means ± SEM. Data were analyzed by Student’s *t*-test or one-way analysis of variance (ANOVA) with Fisher’s LSD test or two-way ANOVA with a mixed-effects model and Bonferroni’s post hoc tests to correct for multiple comparisons, as appropriate. Statistical comparisons were performed using GraphPad Prism 9 software (GraphPad Software, La Jola, CA, USA). Differences with *p* values < 0.05 were considered statistically significant.

## 3. Results

### 3.1. Elevated Renin mRNA in the SFO and PVN of DOCA-Salt Hypertensive Mice

**Figure 1:**
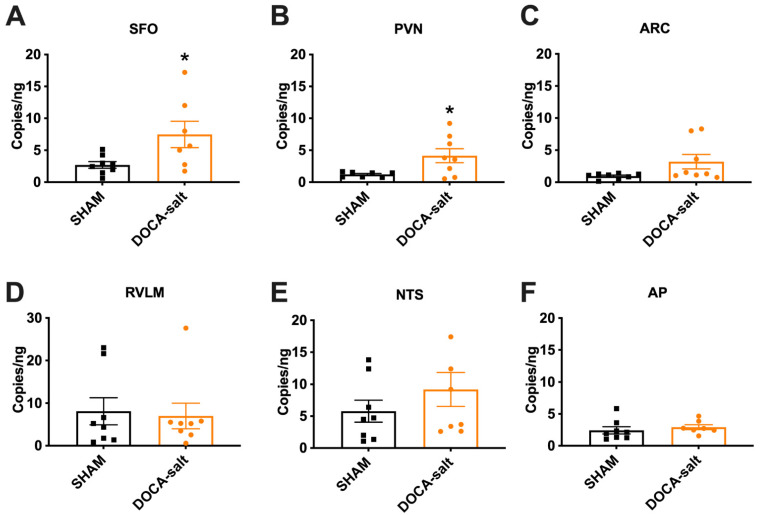
Renin mRNA is upregulated in the SFO and PVN of DOCA-salt–treated mice. (**A**–**F**) Total renin levels were detected in the subfornical organ (SFO), paraventricular nucleus of hypothalamus (PVN), arcuate nucleus (ARC), rostral ventrolateral medulla (RVLM), nucleus tractus solitarius (NTS), and area postrema (AP) using ddPCR. * *p* < 0.05 versus sham treatment (Student’s *t* test); n = 7–9 mice/group.

In the brain, total renin mRNA is composed of both renin-a and renin-b. Previously, studies showed that 21 days of DOCA-salt inhibits renin-b while increasing renin-a expression, which contributes to hypertension development [21,30]. These differential expression patterns were evaluated in whole-brain homogenates, and thus provided no information about spatial distribution. Here, we examined expression in specific brain regions using digital droplet PCR. Because the mRNA sequence that distinguishes between renin-a and renin-b is extremely small [16,17], we examined total renin mRNA level as a surrogate following DOCA-salt treatment. Total renin mRNA levels were significantly increased in the subfornical organ (SFO; *p* = 0.03) and paraventricular nucleus (PVN; *p* = 0.027) of DOCA-Salt mice compared with Sham mice (Figure 1A,B). There were no significant differences in other brain regions examined, including the arcuate nucleus (ARC), rostral ventral lateral medulla (RVLM), nucleus tractus solitarius (NTS), and area postrema (AP) (Figure 1C–F). These data indicate that mRNA for total renin, and potentially that for renin-a, is elevated in the SFO and PVN of DOCA-salt hypertensive mice. 

### 3.2. Cellular Characterization of Renin mRNA in the Brain

**Figure 2:**
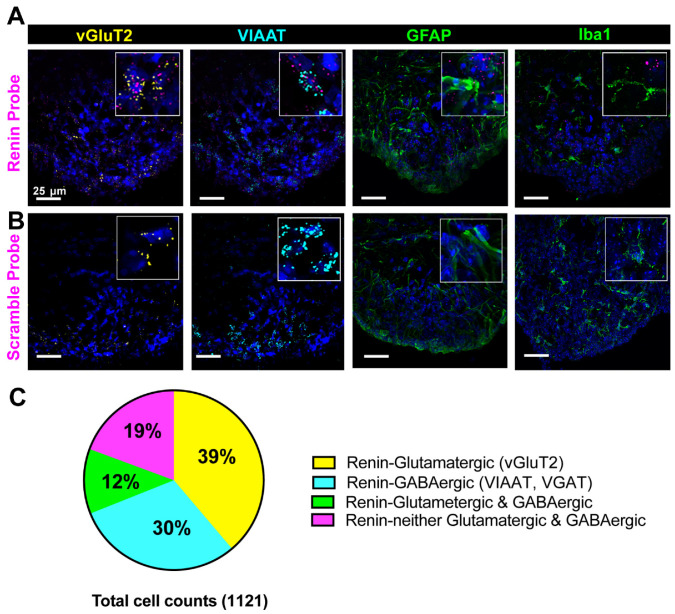
Cell type expression of renin in the SFO. Renin mRNA and mRNAs for nucleus-specific GABAergic and glutamatergic neuron markers were detected in the mouse brain (n = 4 mice) using RNAScope in situ hybridization. Cells were immunolabeled for astrocytes (GFAP) and microglia (Iba1) markers. (**A**) Representative confocal images of renin mRNA in the SFO and (**B**) scrambled renin probe (negative control). Magenta, renin; yellow, glutamatergic neurons (vGluT2); cyan, GABAergic neurons (VIAAT); green, astrocyte marker (GFAP) or microglia marker (Iba1). Tissues were counterstained with DAPI (blue). (**C**) Summary data showing the cellular distribution of renin mRNA in GABAergic and/or glutamatergic neurons. Abbreviations: SFO, subfornical organ; vGluT2, vesicular glutamate 2; VIAAT, vesicular inhibitory amino acid transporter; GFAP, glial fibrillary acidic protein; Iba1, ionized calcium binding adaptor molecule 1.

RNAscope in situ hybridization allows specific, sensitive fluorescent visualization of individual mRNA strands, each of which is represented by a single punctum at the cellular level. The SFO is a key circumventricular organ of the brain that is important for BP regulation [31]. Focusing on the SFO, we further examined the cellular characteristics of renin mRNA. As shown in representative images (Figure 2A,B), renin mRNA was detected in both glutamatergic and GABAergic neurons. Specifically, quantitative analyses (Figure 2C) showed that 43% of renin mRNA-containing neurons were glutamatergic (vGluT2-positive) and 35% were GABAergic (VIAAT/vGAT-positive). Interestingly, ~17% of renin mRNA-containing neurons expressed both vGlut2 and VIAAT, whereas 22% were neither glutamatergic nor GABAergic. We did not detect any co-localization of renin mRNA puncta with astrocyte (GFAP) or microglia (Iba1) markers. 

### 3.3. Deletion of Renin-a in the SFO Attenuates DOCA-Salt–Induced Hypertension 

**Figure 3:**
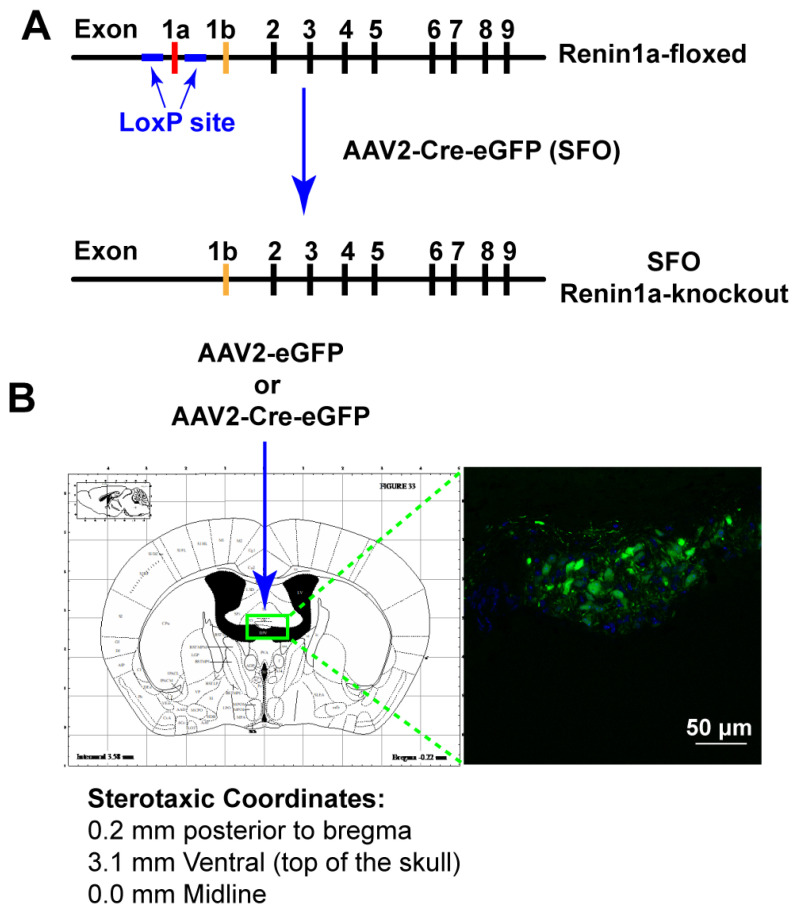
Characterization of SFO-targeted renin-a–KO mice. (**A**) Schematic illustrating Cre-loxP recombination in renin-a–floxed mice. (**B**) Schematic brain atlas and coordinates showing injection sites for AAV2-Cre-eGFP or AAV2-eGFP into the SFO and a representative image of eGFP expression in the SFO.

Activation of the RAS in the SFO leads to increased BP and hypertension [32,33]. To determine whether endogenous renin-a within the SFO contributes to the development of DOCA-salt hypertension, we knocked down renin-a by delivering AAV2-Cre-eGFP into the SFO of renin-a–floxed mice; AAV2-eGFP was used as a control (Figure 3A). A schematic showing stereotaxic coordinates and representative images of SFO targeting is presented in Figure 3B. 

**Figure 4:**
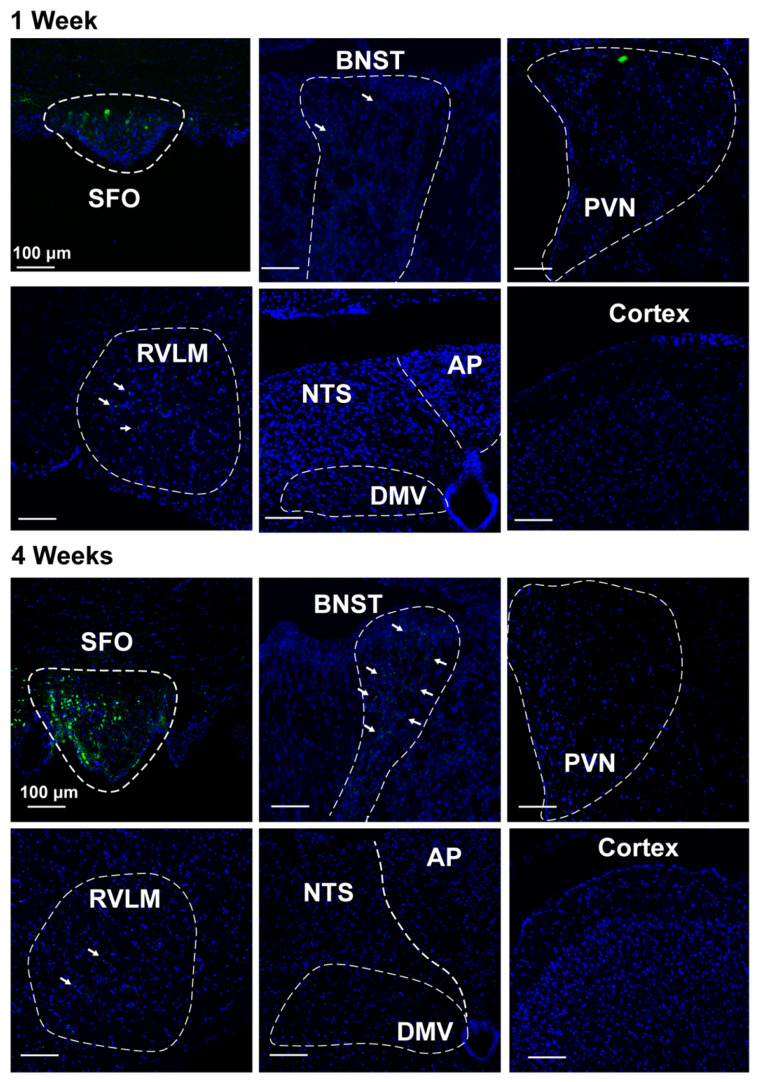
SFO-targeted AAV2-eGFP expression. Images of the SFO, cortex, BNST, PVN, RVLM, DMV, and NTS from mice that received an SFO injection of AAV2-eGFP 1 and 4 weeks after injections. eGFP expression (green) was detected in the cell bodies of SFO, but not in the bed of nucleus stria terminalis (BNST), paraventricular nucleus of the hypothalamus (PVN), rostral ventrolateral medulla (RVLM), nucleus tractus solitarius (NTS), dorsal motor nucleus of the vagus (DMV), area postrema (AP), or cortex. White arrows indicate visible projections (green dots, eGFP) to the BNST and RVLM. No obvious eGFP was observed in other brain regions examined.

To validate the specificity of targeted virus injections, we imaged the SFO 1 and 4 weeks after AAV2-eGFP injection. As shown in representative images (Figure 4), most SFO tissue expressed the eGFP reporter in cell bodies, whereas other key brain regions that are important for BP regulation did not, supporting specific targeting of the SFO using this approach. Four weeks after AAV2 delivery, we observed some visible projections, mostly to the bed nucleus of the stria terminalis (BNST) and RVLM (Figure 4, white arrows), but no cell bodies. The projections were not very robust in brain nuclei downstream of the SFO, likely due to characteristics of the AAV2-eGFP virus that make it less than ideal for visualizing projections. Importantly, however, our data indicate that AAV2 did not infect other brain regions, based on an examination of cell bodies, confirming specific targeting of the SFO. 

**Figure 5:**
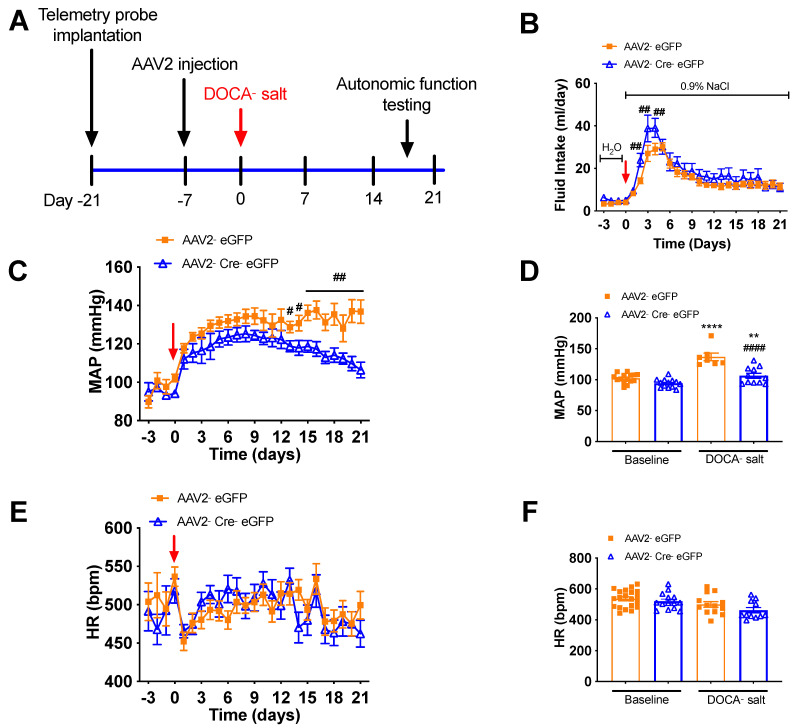
Renin-a deletion in the SFO attenuates DOCA-salt–induced hypertension. Renin-a–floxed mice were injected in the SFO with either AAV2-eGFP or AAV2-Cre-eGFP. BP and HR were monitored by telemetry for 3 days before and 21 days after DOCA-salt treatment. (**A**) Experimental protocol for telemetric probe implantation, virus delivery, and DOCA-salt treatment. (**B**) Fluid intake of mice before and during 21-day DOCA-salt treatment (n = 8–10 mice/group). ^#^ *p* < 0.05, ^##^ *p* < 0.01 versus AAV2-eGFP (two-way ANOVA with mixed-effects model, Fisher’s LSD test). (**C**) Continuous mean arterial pressure (MAP) recordings before and during 21-day DOCA-salt treatment (n = 15–18 mice/group). ^##^ *p* < 0.01 versus AAV2-eGFP (two-way ANOVA with mixed-effects model, Fisher’s LSD test). (**D**) Endpoint MAP at 21 days DOCA-salt treatment (n = 7–15 mice/group). ** *p* < 0.01, **** *p* < 0.0001 versus baseline AAV2-eGFP; ^####^ *p* < 0.0001 versus AAV2-eGFP DOCA-salt (one-way ANOVA with Fisher’s LSD test). (**E**) Continuous HR recording before and during 21-day DOCA-salt treatment (n = 11–20 mice/group). (**F**) Endpoint HR at 21 days DOCA-salt treatment (n = 11–20 mice/group).

**Figure 6:**
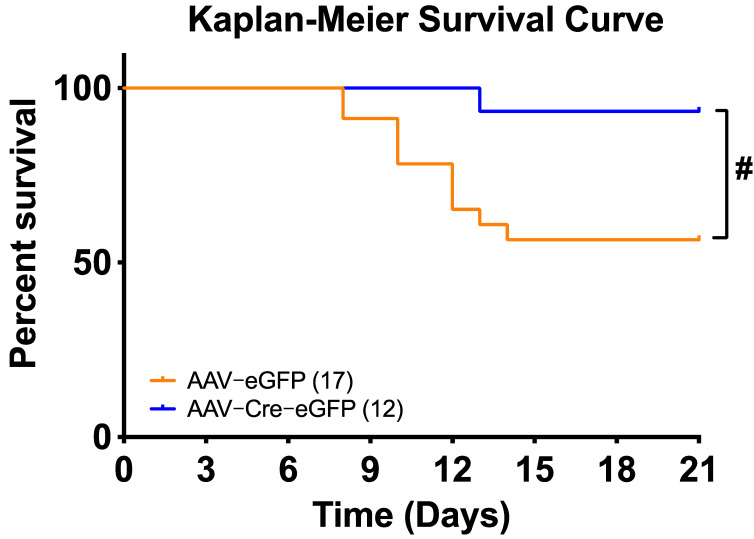
Renin-a ablation in the SFO improves survival rate following DOCA-salt treatment. Kaplan–Meier survival curves during 21-day DOCA-salt treatment. ^#^ *p* < 0.05 versus AAV2-eGFP DOCA-salt (one-way ANOVA with Fisher’s LSD test).

Figure 5A illustrates the experimental protocol for virus delivery and DOCA-salt treatment. At baseline, 24-h BP and HR recorded telemetrically in conscious, freely moving mice were similar between animals that received AAV2-eGFP and those that received AAV2-Cre-eGFP (Figure 5), suggesting that deletion of renin-a in the SFO does not affect baseline BP or HR. Following DOCA-salt treatment, we found no overall difference in saline intake between renin-a–knockout (KO) and control mice (Figure 5B), indicating that both groups of mice ingested the same amount of salt throughout the experiments. Interestingly, however, on days 2, 3 and 4 of DOCA-salt treatment, renin-a–KO mice drank slightly higher amounts of saline. BP in control mice (AAV2-eGFP group) continued to rise following DOCA-salt treatment, and these mice became hypertensive by the end of the protocol compared with their own baseline; importantly, at the end of the 21-day DOCA-salt treatment, BP was significantly lower in mice that received AAV2-Cre compared with controls (Figure 5C,D). Renin-a knockout in the SFO did not completely normalize BP compared with its own baseline (Figure 5D). In terms of HR, two-way ANOVAs revealed no significant differences throughout the protocol between mice that received AAV2-Cre injections in the SFO and those that received control virus (Figure 5E,F). Interestingly, SFO ablation of renin-a significantly improved the survival rate of mice treated with this relatively high dose of DOCA-salt (Figure 6), suggesting a potential beneficial effect of renin-a deletion, although future studies are required to understand causes of mortality and underlying mechanisms. 

**Figure 7:**
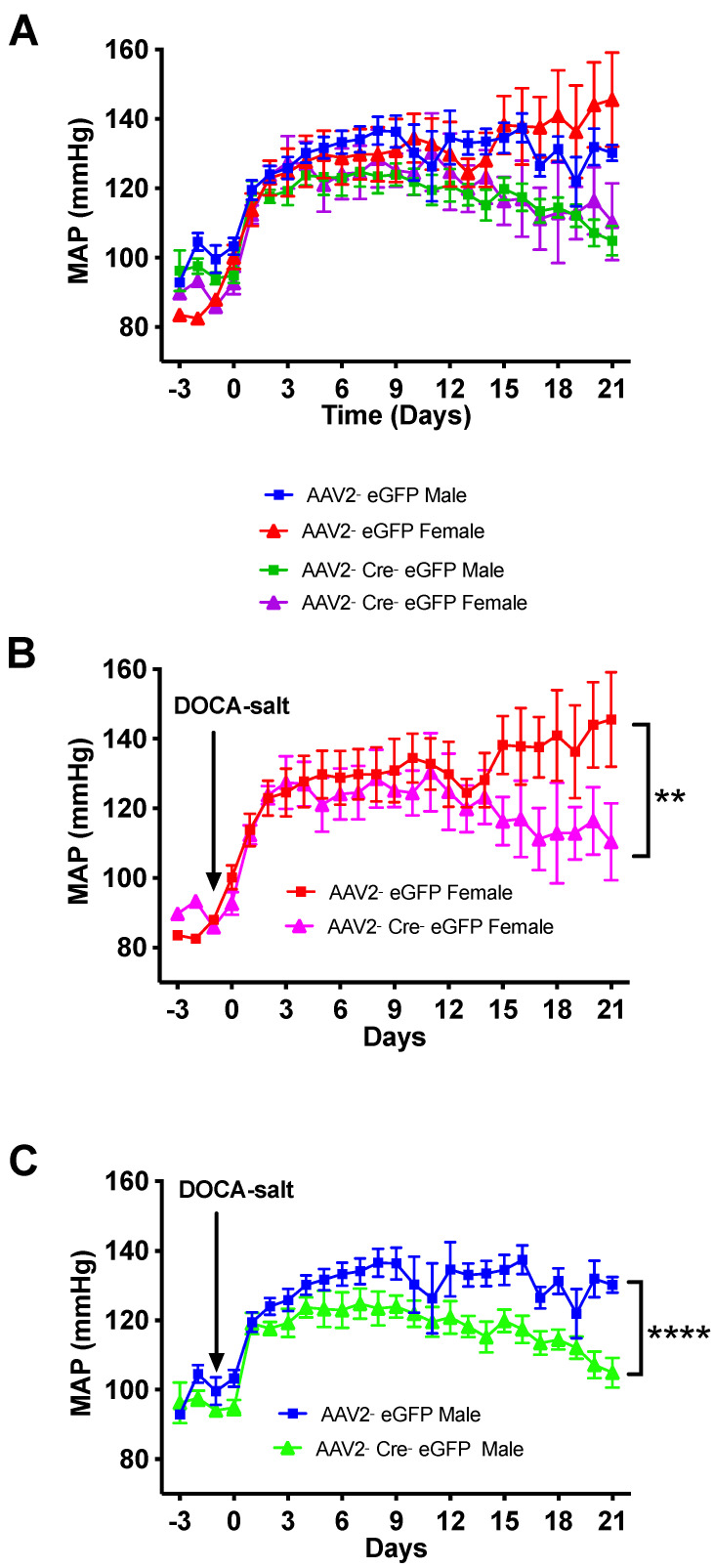
No sex differences in the severity of hypertension. Renin-a–floxed mice were injected into the SFO with either AAV2-eGFP or AAV2-Cre-eGFP. BP was monitored by telemetry for 3 days before and 21 days after DOCA-salt treatment. Continuous mean arterial pressure (MAP) recording before and during 21 days of DOCA-salt treatment, separated into (**A**) all males and females (n = 15–16 mice/group), (**B**) females only (n = 5 mice/group), and (**C**) males only (n = 10–11 mice/group). ** *p* < 0.01, **** *p* < 0.0001 versus AAV2-eGFP DOCA-salt (two-way ANOVA with Fisher’s LSD test).

Distinct sex differences in the incidence and severity of hypertension are well established in humans and animal models of hypertension [34,35,36,37,38]. To determine whether effects of renin-a deletion in mice display sex dimorphisms, we segregated BP data into males and females and found that both male and female mice developed hypertension similarly following DOCA-salt treatment (Figure 7). In addition, the beneficial effect of renin-a deletion in the SFO on hypertension was similar in both male and female mice (Figure 7). 

### 3.4. Deletion of Renin-a in the SFO Improves Autonomic Function in DOCA-Salt Hypertensive Mice

**Figure 8:**
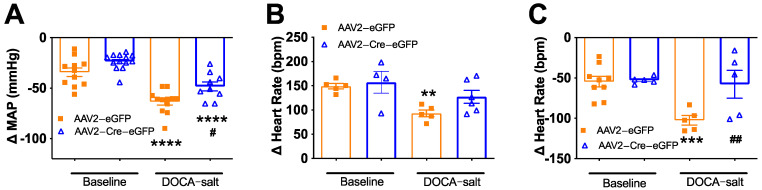
Renin-a deletion in the SFO improves autonomic function in DOCA-salt hypertensive mice. Autonomic function was assessed by intraperitoneal injection of the ganglionic blocker chlorisondamine (6 mg/kg), muscarinic receptor blocker methylatropine (1 mg/kg), or β-adrenergic receptor blocker propranolol (5 mg/kg). (**A**) Reduction in BP response to chlorisondamine, indicative of the neurogenic contribution to BP (n = 9–13 mice/group). (**B**) Increase in HR response to methylatropine, indicative of cardiac parasympathetic tone (n = 4–6 mice/group). (**C**) Reduction in HR response to propranolol, indicative of cardiac sympathetic tone (n = 5–9 mice/group). ** *p* < 0.01, *** *p* < 0.001, **** *p* < 0.0001 versus baseline; ^#^ *p* < 0.05, ^##^ *p* < 0.01 versus AAV2-eGFP DOCA-salt (one-way ANOVA with Fisher’s LSD test).

To determine the contribution of neurogenic mechanisms to the reduced BP in SFO-renin-a–KO mice, we evaluated the magnitude of the BP reduction in response to the ganglionic blocker, chlorisondamine. As shown in Figure 8A, at baseline, the BP response to chlorisondamine was similar between mice that received AAV2-Cre and those that received control virus. DOCA-salt treatment for 21 days significantly increased the magnitude of the BP reduction in response to ganglionic blockade in controls compared with corresponding baseline values, suggesting increased neurogenic hypertension. In contrast, the reduction in BP at the end of DOCA-salt treatment was attenuated in SFO-prorenin–KO mice compared with that in controls. These findings indicate that neurogenic hypertension is decreased in these mice, although we note that the neurogenic pressor response was not completely restored to baseline levels by prorenin knockout in the SFO. 

We further assessed cardiac parasympathetic and sympathetic tone by blocking muscarinic receptors and beta-adrenergic receptors with methylatropine and propranolol, respectively, and monitoring HR changes using telemetry. DOCA-salt treatment significantly reduced the HR response to methylatropine in control mice, indicating a reduction in cardiac parasympathetic tone in DOCA-salt hypertension, whereas there was no significant reduction in HR in SFO-prorenin–KO mice (Figure 8B). On the other hand, the HR response to propranolol was increased in control mice treated with DOCA-salt, suggesting elevation of cardiac sympathetic tone; importantly, the HR response to propranolol was significantly lower in mice in which prorenin was ablated in the SFO, indicating lower cardiac sympathetic tone (Figure 8C). Taken together, these data indicate that prorenin knockout in the SFO reduces neurogenic hypertension and improves cardiac sympathetic and parasympathetic tone in DOCA-salt hypertension. 

### 3.5. Renin-a Ablation in the SFO Prevents Upregulation of NOX2 in DOCA-Salt Hypertension

**Figure 9:**
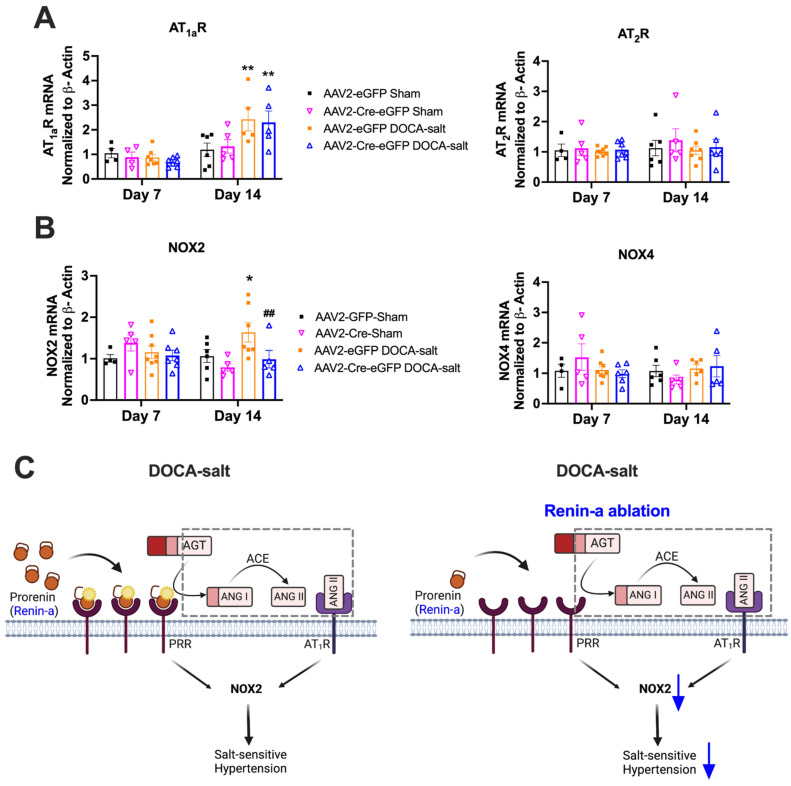
Renin-a deletion in the SFO prevents upregulation of NOX2 during DOCA-hypertension. mRNA expression of target genes measured in SFO samples from DOCA-Salt or Sham mice on days 7 and 14 of treatment. (**A**) Quantitative PCR analysis of mRNA expression of Ang II receptors (AT_1a_R and AT_2_R) and (**B**) NAD(P)H oxidases (NOX2 and NOX4). (**C**) Mechanistic hypothesis for the effect of SFO ablation of renin-a on salt-sensitive hypertension (illustration created using Biorender); pathways in dotted gray square were not directly tested in this study. * *p* < 0.05, ** *p* < 0.01 versus baseline AAV2-eGFP Sham; ^##^ *p* < 0.01 versus AAV2-eGFP DOCA-salt (one-way ANOVA with Fisher’s LSD test). Abbreviations: AT_1a_R, angiotensin II type 1a receptor; AT_2_R, angiotensin II type 2 receptor; NOX2, NADPH oxidase isoform 2; NOX4, NADPH oxidase isoform 4; SFO, subfornical organ.

To identify potential downstream molecular events that may be responsible for the effects of renin-a deletion in hypertension maintenance, we examined the impact of renin-a ablation in the SFO on Ang II type 1a (AT_1a_R) and type 2 (AT_2_R) receptors 7 and 14 days after DOCA-salt treatment (Figure 9A). These two time points were chosen because the effect of renin-a ablation on BP became apparent after the second week of DOCA-salt treatment. AT_2_R expression levels were unchanged among groups at both 7- and 14-day time points, and AT_1a_R expression was unchanged among groups at the 7-day time point. However, AT_1a_R levels in the SFO were significantly increased by DOCA-salt treatment at 14 days, an effect that was not blocked by renin-a deletion (Figure 9A). 

NOX activation is one of the key mechanisms of hypertension development, particularly in the SFO [39,40,41,42]. We thus further examined NOX2 and NOX4, two important NOX isoforms in the brain (Figure 9B). There was no change in NOX2 or NOX4 levels among groups following 7 days of DOCA-salt treatment, or on either NOX isoform at 7- or 14-day timepoints in the Sham group. However, DOCA-salt significantly elevated NOX2, but not NOX4, mRNA levels in the SFO at day 14 of treatment; notably, this NOX2 elevation was prevented by renin-a ablation in the SFO (Figure 9B). These data indicate that renin-a ablation in the SFO attenuates DOCA-salt–induced NOX2 upregulation, suggesting that this upregulation contributes to lowering BP. We propose that renin-a ablation in the SFO attenuates hypertension, possibility by directly reducing PRR activation and/or Ang II formation and thereby reducing AT_1a_R activation, and ultimately attenuates NOX2 activation, as shown in Figure 9C.

## 4. Discussion 

Prorenin is traditionally viewed as precursor of renin with minimal ability to cleave angiotensinogen to angiotensin I [43,44]. We recently reported that exogenous administration of prorenin into the CNS induces a marked elevation in BP that is dependent on the neuronal PRR, indicating the potential functional importance of CNS prorenin in BP regulation [10]. However, the role of endogenous brain prorenin (or renin-a) in BP regulation and the development of hypertension has remained undefined. Taking advantage of new techniques and a novel renin-a–floxed mouse strain, we here report the following important findings: (1) renin mRNA is present locally in brain regions including the SFO, PVN, NTS, DMV, and RVLM; (2) renin mRNA is detected in several types of neurons, but not in astrocytes or microglia, and in the SFO, is present in both glutamatergic and GABAergic neurons, although some renin-expressing cells are neither glutamatergic nor GABAergic; (3) total renin mRNA level is elevated in the SFO and PVN of DOCA-salt hypertensive mice; (4) ablation of renin-a specifically in the SFO attenuates DOCA-salt–induced hypertension; (5) renin-a ablation reduces neurogenic hypertension and improves cardiac sympathetic and parasympathetic tone, suggesting the functional importance of endogenous SFO prorenin/renin-a in hypertension; and (6) mechanistically, renin-a ablation attenuates DOCA-salt-induced hypertension, likely in part by preventing NOX2 upregulation. 

The first key finding of the current study is the direct evidence for renin mRNA expression in various cardiovascular regulating nuclei, including the SFO, PVN, RVLM, and NTS/DMV. These findings extend those of Lavoie et al., who previously reported renin promoter-driven expression of eGFP in the brains of transgenic reporter mice (REN-1C/eGFP mice) [11]. Using eGFP as reporter, they showed that renin promoter activity was detectable in several regions of the brain, including the cerebellum, hippocampus, RVLM and SFO, among others. Consistent with our findings, they also reported renin promoter activity in neuronal cells but not in astrocytes. Additional work by Lavoie et al. using dual-reporter transgenic mice—REN-1C/eGFP together with β-galactosidase driven by the human AGT promoter (hAGT/β-gal)—revealed that cells with renin-promoter activity are in close proximity to AGT-expressing cells in multiple regions of the brain, including the RVLM and SFO, which are known to control cardiovascular function [12]. Our work is congruent with these previous reports of the presence of renin promoter activity in neurons among cardiovascular regulatory brain nuclei based on the use of reporter systems. In the SFO, renin mRNA is present in both glutamatergic and GABAergic neurons, but not in astrocytes or microglia. Interestingly, some renin-expressing cells are both glutamatergic and GABAergic, whereas other renin-expressing cells are neither glutamatergic nor GABAergic, indicating a complex renin mRNA expression pattern in the SFO. 

Despite previous literature showing renin mRNA expression [30] or enzymatic activity [45] and an increase in prorenin protein levels in homogenized brain tissues under pathophysiological conditions [1,10], the cellular and regional localization, as well as the physiological significance, of brain endogenous prorenin are incompletely understood. For example, a recent study by van Thiel et al. showed a reduction (~60%) in total renin activity in the brain after clearing the cerebral vasculature of blood, prompting the authors to suggest that there is no brain RAS [46], a conclusion that ignores numerous studies supporting the presence of a functional brain RAS [1,10,15,25,47,48,49,50,51]. We note here that, although renin/prorenin levels were significantly decreased after blood was removed from the brain vasculature in this latter study, renin/prorenin was still in the detectable range for most brain regions studied, clearly demonstrating the presence of endogenous renin and prorenin in the brain. Furthermore, a very early study by Genain et al. [45] detected both renin activity and angiotensinogen expression in the CNS, and showed that sodium chloride deprivation increased renin activity in the olfactory bulb and anterior pituitary. The authors of this study also controlled for residual plasma contamination by both perfusing rats and evaluating renin activity in brain regions of nephrectomized rats, approaches that collectively showed the presence of renin activity in brain tissues. In the current study, using RNAScope and ddPCR we were able to directly detect renin mRNA in the brain and determine its cellular localization in neurons. 

Another key finding of this study is the functional importance of endogenous SFO renin-a in the maintenance of hypertension. In the CNS, renin is encoded by two alternative transcripts: classical prorenin (also called sREN), encoded by renin-a, and an intracellular form of prorenin (also called icREN) encoded by renin-b [52]. Elegant studies have established the importance of renin-b, or icREN, in the brain. Interestingly, deletion of icREN activates the brain RAS, increasing BP as well as inducing dipsogenic and metabolic effects, possibly through increased renin-a expression or activity [18,19,20,21,22,23,53], suggesting the potential functional importance of renin-a. Focusing on the SFO, we found that renin-a ablation attenuated the maintenance of DOCA-salt–induced hypertension, indicating a regulatory role of SFO renin-a in hypertension. To our knowledge, these findings represent the first demonstration of a functional role for endogenous renin-a/prorenin in salt-sensitive hypertension. The mRNA sequence that distinguishes renin-a mRNA from renin-b mRNA is small. One limitation of this study is that, in our hands, we were unable to detect renin-a and renin-b separately using previously reported primers [21,54]. We note that this renin-a–floxed mice was previously validated by Sigmund’s group [23,24], who generously provide this strain of mouse to our laboratory. Interestingly, renin-a ablation did not prevent the early development of DOCA-salt hypertension but did attenuate hypertension maintenance. One possibility explanation for this effect is that the early phase of DOCA-salt hypertension largely reflects the contribution of humoral actions and volume expansion, whereas the neurogenic component starts to play a greater role 2 weeks after DOCA-salt treatment [55,56]. The other possibility is that, because DOCA-salt treatment was started 1 week after renin-a ablation, renin-a might not yet be sufficiently depleted during the first week of DOCA-salt treatment. Taken together, data from this study demonstrate the functional importance of endogenous renin-a in the SFO in the maintenance of hypertension. 

Activation of the brain RAS, particularly AT_1a_Rs, has been shown in several types of hypertension models, including DOCA-salt hypertension [25,57,58,59,60,61,62]. Here, we sought to understand whether part of the mechanism underlying the actions of renin-a deletion reflects altered regulation of the angiotensin receptors, AT_1a_R and AT_2_R, in the SFO. These experiments showed upregulation of AT_1a_R in the SFO in DOCA-salt hypertension, a finding in agreement with previous reports [63]. However, AT_1a_R remained elevated after 2-weeks of DOCA-salt treatment in mice in which renin-a was ablated in the SFO, indicating that renin-a ablation in the SFO does not regulate BP through alterations in AT_1a_R levels.

NAD(P)H oxidases are critical determinants of BP and hypertension [64]. In the SFO, NOX2 and NOX4 are the most highly expressed NOX homologues [65]. In this context, we found that renin-a deletion prevented DOCA-salt–induced NOX2 upregulation in the SFO. Several pathways have been showed to activate NAD(P)H oxidase in Ang II-dependent or DOCA-salt–induced hypertension, including Ang II/AT_1a_R activation [39,41,65], direct PRR activation [29,39], and enhanced endothelin-1 signaling [66]. Ablation of renin-a in the SFO is expected to reduce de novo Ang II formation and diminish activation of the PRR due to a reduction in prorenin as an endogenous ligand, resulting in attenuated activation of NOX isoforms, as proposed in Figure 9C. One limitation of the current study was the difficulty in measuring the angiotensin peptides in very small brain regions such as the SFO; thus, we were unable to provide direct evidence for the effects of renin-a ablation on angiotensin formation. Nevertheless, evidence for the ability of exogenous prorenin to mediate Ang II formation via the PRR has been reported previously [10,15]. In DOCA-salt–induced hypertension, elevation of NOX2 and NOX4 has been reported in hypothalamic brain regions, including the PVN [67,68]. The current study showed that, in the SFO, NOX2, but not NOX4, was increased in DOCA-salt hypertension, an effect that was blocked by renin-a ablation, indicating the potential importance of NOX2 in this brain region. 

In summary, we report here that renin mRNA is present locally in cardiovascular regulatory regions of the brain and is elevated in DOCA-salt hypertension, specifically in the SFO and PVN. Knockdown of renin-a in the SFO attenuated the maintenance of DOCA-salt–induced hypertension in association with improved autonomic function, indicating a regulatory role of endogenous SFO renin-a in hypertension. Mechanistically, renin-a deletion in the SFO likely attenuates DOCA-salt–induced hypertension, at least in part, by reducing NOX2 activation. We conclude that renin-a in the SFO contributes to DOCA-salt–induced hypertension and is a key functional component of the brain RAS. Future studies are warranted to dissect the functional role and mechanisms of renin-a in various types of neurons. 

## Figures and Tables

**Table 1:** Quantitative real-time PCR Primers.

		5′ to 3′
AT_1a_R	Forward	TCACCAGATCAAGTGCATTTTGA
	Reverse	AGAGTTAAGGGCCATTTTGCTTT
AT_2_R	Forward	TACCCGTGACCAAGTCCTGA
	Reverse	TACCCATCCAGGTCAGAGCA
Beta actin	Forward	CCAGCCTTCCTTCTTGGGTA
	Reverse	AGAGGTCTTTACGGATGTCAACG
NOX 2	Forward	CCCTTTGGTACAGCCAGTGAAGAT
	Reverse	CAATCCCGGCTCCCACTAACATCA
NOX 4	Forward	TGAACTACAGTGAAGATTTCCTTGAAC
	Reverse	GACACCCGTCAGACCAGGAA
Total Renin	Forward	TGCTTGTGGGATTCACAGCCTCTA
	Reverse	TGTGTCACAGTGATTCCACCCACA

## Data Availability

Not applicable.
